# Alterations of MEN1 and E-cadherin/β-catenin complex in sporadic pulmonary carcinoids

**DOI:** 10.3892/ijo.2012.1563

**Published:** 2012-07-20

**Authors:** SERENA VESCHI, ROSSANO LATTANZIO, GITANA MARIA ACETO, MARIA CRISTINA CURIA, SALVATORE MAGNASCO, DOMENICO ANGELUCCI, ALESSANDRO CAMA, MAURO PIANTELLI, PASQUALE BATTISTA

**Affiliations:** 1 Departments of Experimental and Clinical Sciences; 2 Medical, Oral and Biotechnological Sciences and; 3 Drug Sciences, University ‘G. d’Annunzio’-Chieti-Pescara;; 4 Division of Pathology, ‘SS. Annunziata’ Hospital, I-66013 Chieti, Italy

**Keywords:** pulmonary carcinoids, *MEN1*, *CTNNB1*, *TP53*, mutation, β-catenin, E-cadherin, immunohistochemistry

## Abstract

Pulmonary carcinoids, distinct in typical and atypical, represent 2–5% of all primary lung tumors. The aim of this study was to investigate the molecular alterations correlated with the development of this form of neoplasms. A collection of 38 paraffin-embedded apparently sporadic carcinoids was investigated, through a combined study, for protein expression/localization of menin, p53, β-catenin and E-cadherin and for mutational analysis of the *MEN1, TP53* and *CTNNB1* genes. Menin was expressed in 71% of cases, with a prevalent cytoplasmic (c) localization, β-catenin was expressed in 68.4% of cases, of which 36.8% with a membranous (m) and 31.6% with a cytoplasmic localization. Membranous E-cadherin immunoreactivity was detected in 84.2% cases, nuclear p53 expression in 5.3% of cases. Positive correlation was found between c-menin and c-β-catenin expression (rho=0.439, P=0.008). In addition, m-β-catenin showed a positive correlation with both c-β-catenin and E-cadherin expression (rho=0.380, P=0.022 and rho=0.360, P=0.040, respectively). With regard to the E-cadherin/β-catenin complex, we found also a significant positive correlation between c-menin and ‘disarrayed’ β-catenin expression (rho=0.481, P= 0.007). *MEN1* gene variants were characterized in 34% of cases. c-menin was more highly expressed in tumors with MEN1 variants, compared to tumors without *MEN1* variants (P=0.023). Three nucleotide variants of *TP53* were also detected. This study confirms the involvement of the *MEN1* gene in the development of sporadic pulmonary carcinoids, demonstrates the accumulation of menin in the cytoplasm, and indicates that the disarrayed pattern of the complex significantly correlates with c-menin accumulation.

## Introduction

Pulmonary carcinoids (PCs) account for ∼2–5% of all primary lung tumors (1) and on the basis of the histopathological, biological and clinical features are distinct in typical and atypical carcinoids (TCs and ACs respectively) (2). This distinction is based on neuroendocrine morphology, number of mitoses, absence or presence of necrosis and size of primary tumor (3). Most PCs are confined to the main or lobar bronchi (4), however, 10–15% of cases present with regional lymph node metastases, thus they are classified as malignant, albeit low-grade, neoplasms (1). Distant metastases involving liver, bone, adrenal gland and brain occur in 15% of cases (5). The 5-year survival of TCs is 87–100%, whereas ACs have a more aggressive clinical course, with a 5-year survival of 37–71% (4).

PCs are rare and usually occur sporadically. Infrequently (5%) they arise in association with multiple endocrine neoplasia type 1 (MEN1), an autosomal-dominant familial tumor syndrome characterized by a high frequency of endocrine neoplasms (6). The *MEN1* gene is also implicated in the pathogenesis of sporadic PCs, and mutations of this gene have been the first genetic alterations identified in these tumors (6). Somatic *MEN1* mutations have been detected in 35% of bronchial carcinoid tumors (7). Overall, inactivation of the *MEN1* gene by mutation is detectable in ∼47% of sporadic TCs and in ∼70% of sporadic ACs (8). Recently, somatic inactivating mutations in *MEN1* have been also reported in 44% of pancreatic neuroendocrine tumors (9,10). Menin, the protein encoded by the *MEN1* gene, is a component of histone methyltransferase complexes (11–13) and is ubiquitously expressed. It is predominantly a nuclear protein in non-dividing cells, but in dividing cells it is found mainly in the cytoplasm (7). Menin regulates gene transcription, cell proliferation, apoptosis and genomic stability. One of the proteins interacting with menin is β-catenin, an E-cadherin signaling component, that acts as a transcription factor and whose dysregulation has been associated with the development and progression of many solid tumors, including several types of endocrine tumors (14,15). The E-cadherin/β-catenin complex localizes at the cell membrane in essentially all normal and hyperplastic neuroendocrine cells of the lower respiratory tract, giving rise to a membrane-linear immunostaining pattern. The expression of the E-cadherin/β-catenin complex appears conserved in pulmonary neuroendocrine tumors. However, the subcellular compartmentalization of E-cadherin and β-catenin is profoundly heterogeneous in diverse tumor types, and reflects in a differential distribution of the membrane-linear/disarrayed immunostaining pattern ratio (13). Only a minority of lung neuroendocrine tumors show a nuclear translocation of β-catenin, most cases showing a membranous colocalization with E-cadherin. The β-catenin nuclear accumulation appears to be an exclusive feature of a subset of high-grade neuroendocrine tumors (14). Consistently, abnormal cytoplasmic and/or nuclear localization of the E-cadherin/β-catenin complex are independent predictors of lymph node metastasis in ACs (14,15). Finally, LOH and point mutations of the *TP53* locus on chromosome 17p13 have also been detected in 10% of TCs and in 45% of ACs, and were proposed to increase with the severity of the tumor type (16).

However, a comprehensive scenario of the molecular alterations associated with PCs and of their interactions is still missing. Hence, we investigated 38 sporadic PCs for protein expression/localization (nuclear, cytoplasmic and membranous) of menin, p53, E-cadherin and β-catenin combined with mutational analysis of *MEN1, TP53, CTNNB1* genes. Our findings show correlations of specific alterations patterns in different sub-sets, thus suggesting different molecular mechanisms in tumor sub-groups. This may reflect in differential molecular taxonomy of PCs.

## Materials and methods

### Tissue samples

Archived formalin-fixed paraffin-embedded (FFPE) blocks of 38 apparently sporadic PCs consecutively diagnosed between 2001–2008 at the Institute of Pathology, ‘S.S.Annunziata’ Hospital, Chieti, Italy were retrieved. All tumors were reviewed for diagnosis. Cases were classified as TC (30 cases) or AC (8 cases) carcinoid tumors (WHO classification) (2). For each case both tumor and normal tissues were available. The study was reviewed and approved by the ethics committee of the ‘S.S. Annunziata’ Hospital.

### Tissue microarray (TMA) construction and IHC

TMA was constructed by extracting 2-mm diameter cores of histologically confirmed neoplastic area and re-embedding the cores into gridded paraffin blocks, using a precision instrument (Beecher Instruments, Sun Prairie, WI). TMA sections were stained using the anti-Menin polyclonal rabbit antibody (1:350 dilution, 30 min, Bethyl Laboratories Inc., Montgomery, TX). For β-catenin, in order to validate the results of the immunohistochemistry analysis, we used two commercially available mouse monoclonal antibodies raised against the C-terminal domain of β-catenin, clone 17C2 (1:100 dilution, 60 min, Novocastra, Laboratories Ltd., Newcastle, UK) and 14/β-catenin (1:150 dilution, 60 min, BD Transduction Laboratories, San Jose, CA). The anti-E-cadherin (1:50 dilution, 30 min, HECD-1, Zymed Laboratories Inc., San Francisco, CA) and the anti-p53 (1:50 dilution, 30 min, DO7, Novocastra) mouse monoclonal antibodies were also used. Antigen retrieval was performed by microwave treatment at 750 W for 10 min in 10 mM sodium citrate buffer pH 6.0 (S2031, Dako, Glostrup, Denmark), except for sections stained with the anti-p53 antibody that were treated in thermostatic bath at 96°C for 40 min in sodium citrate buffer (Dako) The anti-mouse and the anti-rabbit EnVision kits (Dako) were used for signal amplification, as appropriate. In control sections the specific primary antibody was omitted or replaced with non-immune serum or isotype-matched immunoglobulins.

Immunohistochemical results were evaluated by two patho-logists (M. Piantelli and R. Lattanzio) by consensus without knowledge of the clinicopathologic information. Menin and p53 status were considered positive when ≥1% of the tumor cells were stained. The immunostaining pattern of tumor cells for β-catenin and E-cadherin was defined as arrayed or disarrayed according to the immunohistochemical criteria proposed by Pelosi *et al*(14). Arrayed staining was defined as a membrane-associated, linear pattern of immunoreactivity for β-catenin and E-cadherin, which decorated entire the cell membrane. Disarrayed staining was defined as a membrane staining observed along with variable cytoplasmic accumulation or if a prevalent cytoplasmic staining with only minimal or absent membrane labeling.

### DNA extraction

Representative areas of tumor and normal tissues were identified within hematoxylin-counterstained deparaffinized sections and separated by manual microdissection into 1.5 ml polypropilene vials. For DNA extraction we cut FFPE unsectioned core samples from the interior of the paraffin blocks selecting tumor and surrounding normal lung tissue areas. Tumor and non-tumor DNAs were extracted using the RecoverAll™ Total Nucleic Acid Isolation kit according to the manufacturer’s instructions (Applied Biosystems, Forster City, CA). Purified DNA was easily amplifiable and suitable for denaturing high performance liquid chromatography (DHPLC) analysis.

### Mutational analysis

DHPLC and direct sequence techniques were used to analyze the entire coding sequence of *MEN1* gene, exons 5–8 of *TP53* gene and exon 3 of the *CTNNB1* gene for somatic mutations. DHPLC was performed using the Wave^®^ Nucleic Acid Fragment Analysis system (Transgenomic Inc., San Jose, CA) and sequencing analysis using an ABI PRISM 3100 Genetic Analyzer (Applied Biosystems). Each sample was analysed for somatic nucleotide variants, by sequence comparison of tumor and non-tumor DNA. We analyzed PCR amplicons of DNA extracted from FFPE tissues by DHPLC. Tumor DNAs were analyzed for the entire *MEN1* coding sequence, including intron-exon boundaries, using 13 PCR primer sets for exons 1–10, as previously described (17). We designed also a set of primers to amplify the last part of *MEN1* exon 10 (forward: AACTCGAGCGCCATCAAGC; reverse: GGGCTCAGAGTTGGGGGACTA).

For exons 5–8 of the *TP53* gene nested PCR amplifications were performed using primers previously described (18,19). Direct PCR for exon 3 of the *CTNNB1* gene was carried out using the forward primer designed in our laboratory (forward: TGATTTGATGGAGTTGGAC) and the reverse primer previously reported (15). Tolerability prediction of amino acid changes was tested by SIFT version 2 (available at http://blocks.fhcrc.org/sift/SIFT.html) (20). The fruitfly software (www.fruitfly.org) was used to assess *in silico* predicted effects on splicing of intron nucleotide variants.

### Statistical data analysis

Comparisons between molecular markers were done by the Spearman’s Rho correlation. The independent samples t-test was used to compare the expression of molecular markers in PCs according to *MEN1* gene status. The SPSS program (version 15.0, SPSS Inc., Chicago, IL, USA) was used for statistical analysis. All cited P-values are two-sided; P<0.05 was considered as statistically significant.

## Results

### Immunohistochemistry

**Figure 1:**
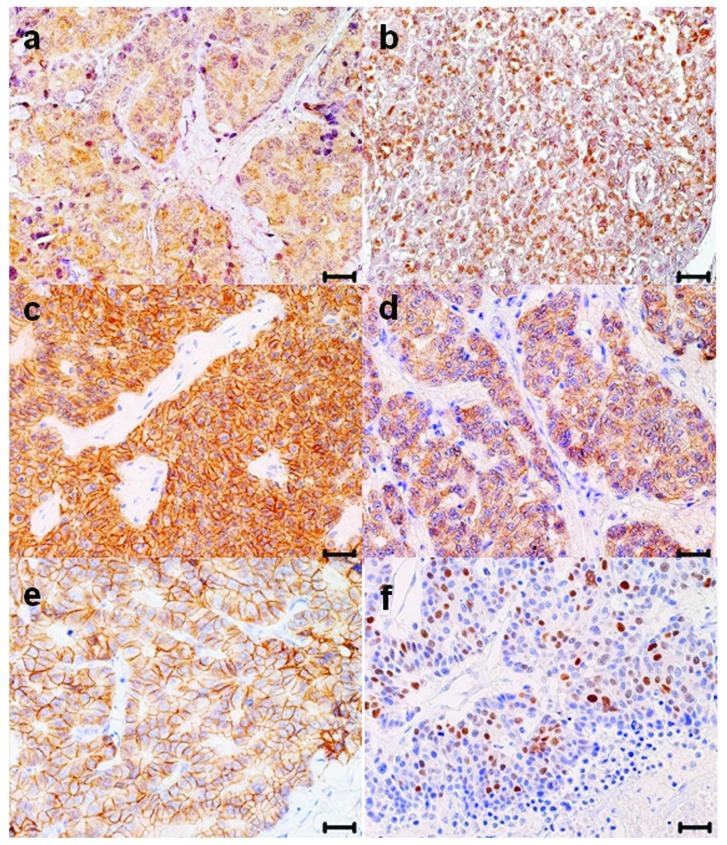
Immunostaining in PCs. Cytoplasmic (a) and nuclear (b) menin staining; membranous staining (c) and prominent cytoplasmic staining (d) for β-catenin; membranous staining for E-cadherin (e); nuclear staining for p53 (f). Original magnification ×40; scale bar, 20 μm.

The results of the IHC analysis for menin, β-catenin, E-cadherin and p53 expression performed on 38 PCs are reported in Table I and examples of specific immunohistochemical stainings are shown in Fig. 1. There were in total 27 menin positive tumors out of 38 (71.0%), of these, 26 out of 38 (68.4%) cases expressed menin in the cytoplasm of tumor cells (c-menin) with a mean value of positive tumor cells of 44.7±6.1 (mean ± SE) and 3 out of 38 (7.9%) tumors, two of which were also c-menin positive, showed specific nuclear immunoreactivity for menin (n-menin) (2.3±2.1).

β-catenin-positive cases were 26 out 38 (68.4%). No differences in the β-catenin immunostaining were observed using the two different monoclonal antibodies. Membranous β-catenin (m-β-catenin) expression was observed in 14 out of 38 (36.8%) cases, whereas cytoplasmic β-catenin (c-β-catenin) was detected in 12 out of 38 (31.6%) cases. Coexpression of m- and c-β-catenin was detected in 6 cases. The mean values of β-catenin-positive tumor cells were 8.0±3.1 for m-β-catenin and 16.7±5.0 for c-β-catenin. β-catenin was not expressed in the nucleus of tumor cells. E-cadherin immunoreactivity was observed exclusively in cell membrane and detected in 32 out 38 (84.2%) cases with a mean ± SE of positive tumor cells of 58.6±6.7. The nuclear expression of p53 (n-p53) was observed in 2 out of 38 (5.3%) cases, with a mean ± SE of tumor positive cells of 2.1±1.9.

Correlations between markers, were analyzed using the Spearman’s coefficient correlation test. A significant positive correlation was found between c-menin and c-β-catenin expression (rho=0.439, P=0.008) (Table IIA). Furthermore, m-β-catenin showed a positive correlation with both c-β-catenin and E-cadherin expression (rho= 0.380, P= 0.022 and rho= 0.360, P=0.040, respectively). With regard to the protein status of the E-cadherin/β-catenin complex, following the criteria suggested by Pelosi *et al*(14) we found a significant positive correlation between c-menin and β-catenin disarrayed expression (rho=0.481, P=0.007) (Table IIB).

### Mutational analysis

DHPLC and direct sequencing analyses were utilized to detect somatic mutations in *MEN1* (entire coding sequence), *TP53* (exons 5–8) and *CTNNB1* (exon 3) genes. *MEN1* gene variants (ENST00000312049) were identified in 13/38 (34%) cases, of which 9/30 TPCs (30%) and 4/8 APCs (50%) (Table III). Variants included the frameshift mutation c.427delC, which introduces a stop signal at codon 184 (p.L143fsX184, case no. 23) and 4 missense variants (p.T541A, case no. 12; p.G99S, case no. 24; p.A216T, case no. 27; p.L89R, case no. 33), whose tollerance of amino acid changes was tested through SIFT Version 2 program. In addition, we found 3 synonymous variants (p.S145S, cases no. 15 and 23; p.A49A, case nos. 29; p.N418N, case nos. 11, 12, 16 and 37 in heterozygosity, cases no. 2, 28 and 33 in homozygosity). Finally, we characterized also a novel intronic variant (c.446-5C>T, IVS2, case nos. 9 and 12) that, according to the *in silico* evaluation (www.fruitfly.org), was not predicted to affect splicing. In Table III the expression of c- and n-menin, n-p53, m- and c-β-catenin and E-cadherin are reported for each *MEN1* mutated case. As shown, 11 out of 13 and 12 out of 13 *MEN1* mutated cases expressed c-menin and E-cadherin, respectively. Nine out of 13 and 7 out of 13 *MEN1*-mutated cases expressed m- and c-β-catenin, respectively, while the co-expression of m- and c-β-catenin were detected in 6 cases, c- and n-menin and n-p53 were co-expressed in only 1 case. Correlating the menin expression levels with the presence or absence of *MEN1* nucleotide variants, we found that c-menin was significantly more expressed in tumors with *MEN1* variants compared to tumors without *MEN1* variants (P=0.023), whereas n-menin does not show a significance when compared with PCs with and without *MEN1* variants (Table IV). No differences were found comparing m- and c-β-catenin, m-E-cadherin and n-p53 expression levels with *MEN1* gene status, although a positive trend in the expression of c-β-catenin marker in *MEN1* mutated cases was also observed (data not shown). Mutational analysis of *TP53* exons 5–8 allowed to identify 3 nucleotide variants (p.A129A, case no. 21; pI255F, case no. 27; p.R213R, case no. 37) in exons 5, 7 and 6 respectively (Table V), i.e., outside *TP53* hotspots of mutations (21). These nucleotide variants are reported in the *IARC TP53* database (http://www-p53.iarc.fr/), where the tolerance of amino acid changes was tested through SIFT version 2 program (http://blocks.fhcrc.org/sift/SIFT.html) and AGVGD (http://agvgd.iarc.fr/). The variant c.763T at codon 255, reported to be deleterious, resulted associated with a high nuclear expression of p53 (68% of positive tumor cells). Finally, the mutational study of exon 3 of the *CTNNB1* gene resulted negative and no nucleotide variants were detected.

## Discussion

In this study we analyzed a series of 38 sporadic PCs for somatic mutations and for protein expression of genes that appear to be implicated in the development and progression of the disease. Combined genetic and IHC findings were used to identify relevant PC sub-groups and to help defining a combined role of genes potentially involved in the pathogenesis of PCs. This was done by evaluating the IHC expression of menin, p53, β-catenin and E-cadherin at subcellular level and by correlating the expression of these markers with the mutational spectra of the *MEN1*, *TP53* and *CTNNB1* genes in both TC and AC tumors.

A significant fraction of tumor samples (34%) harbored *MEN1* gene variants. Most samples showed a cytoplasmic, rather than nuclear, localization of menin. Cytoplasmic localization of menin was observed in tumors with and without *MEN1* variants. Only two cases with nuclear menin immunostaining did not display variants of the *MEN1* gene.

Notably, tumors carrying *MEN1* variants showed significantly higher cytoplasmic expression of the menin, when compared with samples negative for *MEN1* variants. The cytoplasmic localization of menin was detected both in TCs and ACs, the highest fraction being observed in ACs (data not shown), i.e., in cases with higher rate of mitosis. Thus, our data on PCs are in agreement with previous observations in pancreatic endocrine tumors in which a strong association between *MEN1* variants and cytoplasmic localization of menin were observed (10). This is consistent with a role of this oncosuppressor gene in the pathogenesis of sporadic PCs. The analysis of the E-cadherin/β-catenin complex evidenced a significant correlation in the membranous and/or cytoplasmic expression of these two proteins. It is known that the complex plays a crucial role in cell-cell adhesion, and dysregulation of the E-cadherin/β-catenin-dependent adhesion complex has been associated with the development and progression of many solid tumors, including several types of endocrine tumors (14). Thus, we investigated a possible dysregulation of the complex in PCs. We observed β-catenin expression in the majority of tumor cases. However, in a fraction of PCs β-catenin tended to accumulate in the cytoplasm. Notably, we found also a significant direct relationship of c-β-catenin expression with c-menin expression and, following the immunohistochemical criteria suggested by Pelosi *et al*(13) who defined the immunostaining pattern of tumor cells for β-catenin and E-cadherin as arrayed or disarrayed, we searched for possible correlations between these patterns of immunostaining with the other markers analyzed. Intriguingly, we found a significant positive correspondence between the disarrayed expression of β-catenin with the c-menin expression. Consistent with the absence of nuclear β-catenin expression, we did not find mutations of the *CTNNB1* gene (15), further indicating a role of this gene in PC pathogenesis, but not as a driver mutation. It is known that menin regulates gene transcription, cell proliferation, apoptosis and genomic stability and one of the proteins interacting with menin is β-catenin.

Single or multiple *MEN1* sequence variants, consisting in frameshift, missense and silent variants were characterized in 13 out of 38 cases, with a prevalence in ACs compared to TCs. Three of the 4 missense variants characterized in our study were not reported up to now in association with PCs. The p.T541A missense variant is a pathogenetic variant affecting the role of menin in the apoptosis control (22). The missense mutation p.L89R and the polymorphism p.S145S were previously identified as somatic variants also in glucagonoma and parathyroid tumors. The p.L89R detected in case no. 33 was not tolerant using the SIFT Version 2 program and could interfere with menin protein structure and function. Cytoplasmic expression of menin and presence of nucleotide variants of the *MEN1* gene may be indicative of a significant correlation, since cases with *MEN1* nucleotide variants are characterized by a higher number of positive tumor cells expressing menin in the cytoplasmic compartment. It is noteworthy that high cytoplasmic expression of menin has been also detected in most of the cases bearing only the c.1254T polymorphism, suggesting a hypothetical possibility of this silent variant and c-menin accumulation in tumor cells. Thus, our data support the hypothesis that *MEN1* gene variants affect the subcellular localization of the protein causing its accumulation in the cytoplasm. For cases without *MEN1* variants and cytoplasmic menin expression, it may be possible that other genes, partners of *MEN1*, are responsible for the impairment of menin function in PCs. The results are in agreement with other studies on sporadic lung carcinoids (6).

Somatic mutation analysis of exons 5–8 of the *TP53* gene, which encode the DNA-binding region where cancer-associated mutations most frequently occur (23–25), indicates that genetic alterations of this gene may be implicated in the development of a limited fraction of PCs. In this contest, it is relevant to note that the nucleotide variant c.763T at codon 255, detected in an APC with high immunohistochemical expression of p53, is reported to be deleterious in both the SIFT version 2 and AGVGD programs, where the tolerance of amino acid changes was tested. Thus, our results indicate that *TP53*, rather than playing a broad role in PCs (16,26), may operate in specific PC sub-groups, possibly by interacting with menin. Additional studies are required to test this model.

In conclusion, the present study confirmed the implication of *MEN1* gene in the development of sporadic PC. Furthermore, the mutational study of *MEN1* gene, associated with the IHC analysis of menin indicated that tumors displaying *MEN1* nucleotide variant were characterized by a higher accumulation of menin in the cytoplasm and, for our knowledge, this strong association between *MEN1* variants and cytoplasmic localization of menin has not been previously reported in sporadic pulmonary carcinoids, thus representing an interesting finding for this type of tumor.

In addition, this study also indicated that the subcellular compartmentalization of the E-cadherin/β-catenin complex was altered in PCs and the disarrayed pattern of the complex significantly correlated with c-menin accumulation, thus suggesting a possible cooperative role of menin and E-cadherin/β-catenin in the development and/or progression of this endocrine-related tumor.

## Figures and Tables

**Table I:** Expression of immunohistochemical markers in PCs.

	Positive cases		
Marker	n	(%)	Positive tumor cells^a^
c-Menin	26	(68.4)	44.7±6.1
n-Menin	3	(7.9)	2.3±2.1
m-β-catenin	14	(36.8)	8.0±3.1
c-β-catenin	12	(31.6)	16.7±5.0
n-β-catenin	0		
E-cadherin	32	(84.2)	58.6±6.7
n-p53	2	(5.3)	2.1±1.9

a Values given as mean (%) ± SE; c, cytoplasmic; n, nuclear; m, membranous.

**Table II:** Spearman’s correlations between markers in PCs.

A,					
	c-Menin	n-Menin	m-β-catenin	c-β-catenin	E-cadherin
c-Menin					
Rho^a^	1	−0.093	0.196	**0.439**	−0.014
P		0.597	0.259	**0.008**	0.936
n-Menin					
Rho	−0.093	1	−0.077	0.007	0.004
P	0.597		0.661	0.960	0.983
m-β-catenin					
Rho	0.196	−0.077	1	**0.380**	**0.360**
P	0.259	0.661		**0.022**	**0.040**
c-β-catenin					
Rho	**0.439**	0.007	**0.380**	1	0.172
P	**0.008**	0.960	**0.022**		0.340
E-cadherin					
Rho	−0.014	0.004	**0.360**	0.172	1
P	0.936	0.983	**0.040**	0.340	

B,					
	c-Menin	β-catenin (arrayed )	β-catenin (disarrayed)	E-cadherin (arrayed)	E-cadherin (disarrayed)
c-Menin					
Rho^a^	1	−0.479	**0.481**	0.213	0.054
P		0.230	**0.007**	0.318	0.856
β-catenin arrayed					
Rho	−0.479	1	−0.049	−0.127	
P	0.230			0.765	
β-catenin disarrayed					
Rho	**0.481**		1	0.081	0.029
P	**0.007**			0.765	0.922
E-cadherin arrayed					
Rho	0.213	−0.127	0.081	1	
P	0.318	0.765	0.765		
E-cadherin disarrayed					
Rho	0.054		0.029		1
P	0.856		0.922		

a Rho, Spearman’s coefficient correlation; significant correlations (P<0.05) in bold.

**Table III:** Data on allelic variants of *MEN1* gene and correlations with the percentage of positive tumor cells expressing menin, p53, β-catenin and E-cadherin immunostaining.

Case no.	*MEN1* variant	Exon	Effect	c-menin	n-menin	n-p53	m-β-catenin	c-β-catenin	E-cadherin
TC									
2	c.1254T	9	p.N418N	72	0	0	2	54	12
9	c.446-5C>T	IVS2	No effect on splicing	100	0	0	6	63	100
	c.1254C>T	9	p.N418N						
11	c.1254C>T	9	p.N418N	93	0	0	88	92	74
12	c.446-5C>T	IVS2	No effect on splicing	40	0	0	0	0	26
	c.1254C>T	9	p.N418N						
	c.1621A>G	10	p.T541A						
15	c.435C>T	2	p.S145S	98	0	0	4	82	78
16	c.1254C>T	9	p.N418N	100	0	0	0	31	97
23	c.427delC	2	p.L143fsX184	81	0	0	3	0	95
	c.435C>T	2	p.S145S						
24	c.296A	2	p.G99S	0	0	0	0	0	41
37	c.1254C>T	9	p.N418N	0	0	0	7	8	0
AC									
27	c.646G>A	3	p.A216T	42	4	68	0	0	100
28	c.1254T	9	p.N418N	49	0	0	42	0	98
29	c.147T>G	2	p.A49A	82	0	0	1	0	3
33	c.266T>G	9	p.L89R	55	0	0	45	53	96
	c.1254T	2	p.N418N						

c-, cytoplasmic; m-, membrane; n-, nuclear.

**Table IV:** Correlations between Menin expression and *MEN1* gene status.

	Positive tumor cells	
	Mean ± SE^a^	P-value^b^
c-Menin		
PCs with *MEN1* mutations (n=13)	62.5±9.7	0.023
PCs without *MEN1* mutations (n=25)	34.2±7.1	
n-Menin		
PCs with *MEN1* mutations (n=13)	0.3±0.3	0.485
PCs without *MEN1* mutations (n=25)	3.4±3.2	

a Percent of positive tumor cells ± standard error.

b Independent samples t-test.

**Table V:** Data on nucleotide variants of *TP53*, predicted effect on p53 protein and immunohistochemical expression.

						Predicted effect on the protein		
Case no.	Histotype	*TP53* variant	Exon	Effect	Status (Reference)	SIFT	AGVGD	IHC
21	TC	c.387C>T	5	p.A129A	IARC TP53 database	Silent	Silent	0
27	AC	c.763T	7	p.I255F	IARC TP53 database	Deleterious	Deleterious	68^a^
37	TC	c.639A>G	6	p.R213R	IARC TP53 database	Silent	Silent	NA

a Percent of nuclear p53 immunostained cells.
